# Adsorption behavior and performance of ammonium onto sorghum straw biochar from water

**DOI:** 10.1038/s41598-022-08591-5

**Published:** 2022-03-30

**Authors:** Huajie Xu, Bing Wang, Ruohan Zhao, Xiangui Wang, Changbin Pan, Yuting Jiang, Xueyang Zhang, Banggui Ge

**Affiliations:** 1 Moutai Institute, Renhuai, 564500 Guizhou China; 2 College of Resources and Environment Engineering, Guizhou University, Guiyang, 550025 Guizhou China; 3 Key Laboratory of Karst Georesources and Environment, Ministry of Education, Guiyang, 550025 Guizhou China; 4 School of Environmental Engineering, Xuzhou University of Technology, Xuzhou, 221000 Jiangsu China; 5 Kweichow Moutai Co., Ltd, Renhuai, 564500 Guizhou China

**Keywords:** Environmental chemistry, Environmental impact

## Abstract

Sorghum has been widely used for liquor production and brewing, but how to make efficiently utilize sorghum straw (SS) has become an urgent problem. Meanwhile, the wastewater produced by winemaking is typical organic wastewater with a high ammonium concentration. To solve the problem of resource utilization of SS and remove ammonium from water, SS was used to prepare biochar as an adsorbent for ammonium adsorption. Batch adsorption experiments were carried out to study the influencing factors and adsorption mechanisms of ammonium onto sorghum straw biochar (SSB). The results showed that the adsorption capacity of SSB was much higher than that of SS. The SSB pyrolyzed at 300 °C had the highest adsorption capacity. The favorable pH was 6–10, and the optimal dosage was 2.5 g/L. The adsorption process and behavior conformed to the pseudo-second-order kinetic and Langmuir isotherm adsorption models. The maximum ammonium adsorption capacity of SSB at 45 °C was 7.09 mg/g, which was equivalent to 7.60 times of SS. The ammonium adsorption of SS and SSB was mainly chemical adsorption. The regeneration test indicated that SSB had good regeneration performance after three adsorption-regeneration cycles. This work suggests that SSB could be potentially applied to sewage treatment containing ammonium to achieve the purpose of resource recycling.

## Introduction

Ammonium is one of the main forms of nitrogen in wastewater. A large amount of ammonium entering the water environment may result in serious environmental pollution and threaten human health^1,2^. Therefore, effective control and removal of ammonium from wastewater to ensure the quality of the water environment are in need^3^. Nowadays, the commonly used treatments for removing ammonium in water mainly include ion exchange, chemical precipitation, adsorption, biological nitrification/denitrification, physical chemistry, etc.^4–8^. Among them, the adsorption method is considered to be the most promising treatment method. It has the advantages of small equipment area, high removal efficiency, simple process, and renewable adsorbent^9^. However, there are many kinds of adsorbents with different adsorption effects^10–12^. Although some adsorbents have good adsorption effects (such as activated carbon, clay minerals, carbon nanotubes, and graphene), the cost is higher than biochar prepared by straw waste. For example, the activation temperature of activated carbon is high, and the activation process is more complicated than biochar^13^. Therefore, the research and development of a low-cost and high-efficiency adsorbent have become a hot spot in this field.

As one of the main raw materials for liquor making, the planting area of sorghum has been increased with the increase of liquor production, resulting in a large amount of sorghum straw (SS) waste. In 2019, the yield of sorghum in China had reached 7.227 million tons. According to the ratio of sorghum to SS, it was estimated that the production of SS could reach 9.395 million tons^14^. Currently, SS resource utilization methods mainly include straw returning to the field, animal feed, straw energy, and straw substrate, etc. Due to the limitation of technical level, economy and market acceptance, the utilization rate of SS is still relatively low, with only a few parts of them are used as resources, and most of them are disposed of through open burning. It not only causes a waste of resources but also results in pollution to the rural natural environment. Therefore, how to make full use of sorghum stalks as resources has become a challenge.

Biochar refers to a class of highly aromatic insoluble solid substances produced by pyrolysis (generally the carbonization temperature < 700 °C) and carbonization of organic materials such as crop straws, wood materials, and livestock manure under limited or without O_2_ conditions^15,16^. The well-developed porous structure and relatively large specific surface area (SSA) of biochar make it have good adsorption capacity^17^. Therefore, the application of biochar in environmental remediation has attracted much attention^16^. Biochar has been increasingly used as an adsorbent for pollutants such as heavy metals^18–21^, chlorofluoro^22^, organic pollutants^23,24^, phosphate^25–28^, and ammonium, etc.^2,28–32^. Cui et al. (2016) studied the adsorption effect of biochar prepared from six wetland biomass at 500 °C on ammonium and found that the canna biochar had the largest adsorption capacity (5.60 mg/g)^29^. Huang et al. (2020) used clay/biochar composites to adsorb ammonium in water and found that the adsorption process was more in line with the pseudo-second-order kinetic model and Freundlich isotherm adsorption model^33^. When Xue et al. (2019) used food waste-based biochar to adsorb ammonium in water, they found that the Langmuir equation was fit better to the adsorption behavior, and the adsorption of corn stalk biochar to ammonium was a spontaneous exothermic process. The maximum adsorption capacity was 7.174 mg/g^30^. Wang et al. (2020c) used FeCl_3_ and HCl to modify wheat straw biochar, and found that the ammonium adsorption capacity was improved by 14%^34^. Although there have been many studies on the treatment of ammonium in wastewater using biochar from different feedstocks^2,29,30,35^, few studies on the use of SS derived biochar to remove ammonium in water have been reported. The use of biochar prepared by SS can not only effectively adsorb pollutants, but also realize the resource utilization of straw waste. Therefore, the study of sorghum straw biochar (SSB) is in need.

As a kind of special biomass straw with extensive sources, SSB is supposed to be an adsorbent on ammonium in water. The main contents of this work are to (1) prepare and characterize SSB; (2) explore the influencing factors and adsorption mechanisms on ammonium in water with SSB, and (3) evaluate its adsorption and regeneration performance. This work could provide a theoretical basis for the potential application of SSB to absorb ammonium in wastewater and solve the resource utilization of SS.

## Material and methods

### Chemicals and materials

Main reagents: Nessler's reagent, ammonium chloride, potassium sodium tartrate, sulfuric acid, hydrochloric acid, sodium hydroxide, sodium carbonate, sodium bicarbonate, sodium ethoxide, etc. The above reagents are all analytical pure. Ultrapure water is used as test water. The ammonium stock solution is prepared with ammonium chloride to 1000 mg/L, and then diluted to the required ammonium concentration for different tests.

Main instruments: Scanning Electron Microscope (JSM-6610 LA, JEOL, Tokyo, Japan) (SEM). Fourier Transform Infrared Spectrometer (FTIR) (IRAffinity-1, Shimadzu, Japan). Pore Size and SSA Analyzer (Kubox1000, Beijing Builder Electronic Technology Co., Ltd). UV Spectrophotometer (UV-8000ST, Shanghai Yuanxi Instrument Co., Ltd). High-temperature tube furnace (SG-GL1200K, Shanghai Institute of Optics and Fine Mechanics).

### Preparation and characterization of biochar

SS were collected from the organic sorghum base in the suburb of Renhuai City, Guizhou Province, which was obtained permission from the landowner (Supplementary materials). This experiment was carried out in accordance with the national standard from China entitled "Wood charcoal and test method of wood charcoal"^36^. The preparation of SSB was following previous methods^37^. After drying at 80 °C for 24 h, the SS was cut into 1 ~ 3 mm and then put into the tube furnace. At a heating rate of 2.5 °C/min, the carbonization temperature ranged from 300 to 600 °C under the condition of introducing N_2_ and then kept at the target carbonization temperature for 30 min. After cooling, it was ground and passed through a 100–200 mesh sieve to obtain uniform biochar. Biochar prepared at temperatures of 300, 450, and 600 °C was labeled as SSB300, SSB450, SSB600.

Boehm titration method was used to determine the content of acidic oxygen-containing functional groups of biochar^38^. Biochar morphology characteristics were scanned by SEM. The infrared spectrum of biochar was analyzed by FTIR. SSA, total pore volume, and average pore diameter of biochar were determined by BET-N_2_.

### Adsorption test

The concentration of ammonium was measured by Nessler’s reagent method. Three parallel samples were adopted to control the quality of the analysis process. 50 mL PE centrifugal tubes were used to conduct all the adsorption experiments.

#### Adsorption influencing factors test

0.1000 g of SSB300, SSB450, SSB600, and SS were weighed into test tubes. Then 40 mL ammonium solution (concentration of 50 mg/L, pH = 7.0) was added and shaken at 25 °C for 1440 min. After shaking, the ammonium concentration of the supernatant was filtered and analyzed to obtain the biochar with optimal carbonization temperature for subsequent experiments. The optimal adsorbent experiment was selected in the range of 0.0250, 0.0500, 0.1000, 0.1250, 0.1500, 0.1750, and 0.2000 g. The effect of solution pH on the adsorption of ammonium was conducted by adjusting the solution pH from 2.0 to 12.0 by 0.1 mol/L NaOH and 0.1 mol/L HCl.

#### Adsorption kinetics and thermodynamic test

Sorption kinetics was examined at 25 °C with an interval time of 15, 30, 60, 120, 240, 480, 960, and 1440 min at the optimal adsorption conditions. Sorption isotherms were carried out by varying concentrations ranged from 0 to 250 mg/L at 25, 35, and 45 °C shaken for 24 h.

#### Regeneration test

After reaching adsorption equilibrium, SS and SS300 were filtered out. Then 0.1 g SS and SS300 were put into 50 mL test tubes respectively and 40 mL of HCl and H_2_SO_4_ solutions were added (with a concentration of 0.1 mol/L)^39^. After shaking for 1440 min at 25 °C, the samples were filtered and dried at 103 °C, and the above adsorption-regeneration tests were repeated several times.

## Results and discussions

### Physicochemical properties of biochar

**Figure 1:**
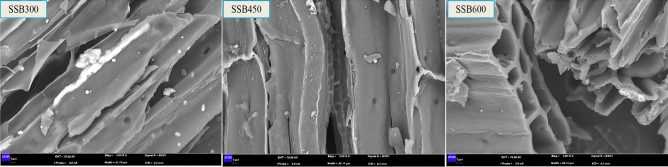
Scanning electron micrograph of biochar at different carbonization temperatures.

The scanning electron microscope images of different biochar are shown in Fig. 1. The increasing carbonization temperature brings more obvious block layering and increases pore structure to biochar. The higher temperature leads to more organic matter decomposing in SS, which makes the biochar appear many micropores in the structure, resulting in the loose layering of the biochar^40^. It shows that high carbonization temperature is beneficial to improve the SSA of biochar. From the physicochemical properties of SSB in Table 1, SSB300 has the highest average pore volume. Affected by the total pore volume, SSB600 has the highest SSA, and SSB300 has the lowest. The result coincides with the inference of the scanning electron microscope image.

**Table 1:** The physicochemical properties of sorghum straw biochar.

Biochars	Acidic oxygen-containing functional groups (mmol/g)				SSA (m^2^/g)	Total pore volume (cm^3^/g)	Average pore size (nm)
	Carboxyl	Lactone group	Phenolic hydroxyl	Carbonyl			
SSB300	1.183	3.02	3.21	8.19	1.41	0.008	11.10
SSB450	0.716	1.99	2.27	5.86	43.51	0.036	1.67
SSB600	0.591	1.81	1.87	4.62	66.74	0.068	2.04

From the analysis data in Table 1, SSB300 has the highest content of carboxyl, lactone, phenolic hydroxyl and carbonyl group, followed by SSB450 and SSB600. It is proved that the acidic functional groups can characterize hydrophilicity and ion exchange capacity gradually decrease with the increase of carbonization temperature^41^. Therefore, from the perspective of acidic oxygen-containing functional groups, SSB300 is more conducive to adsorption.

**Figure 2:**
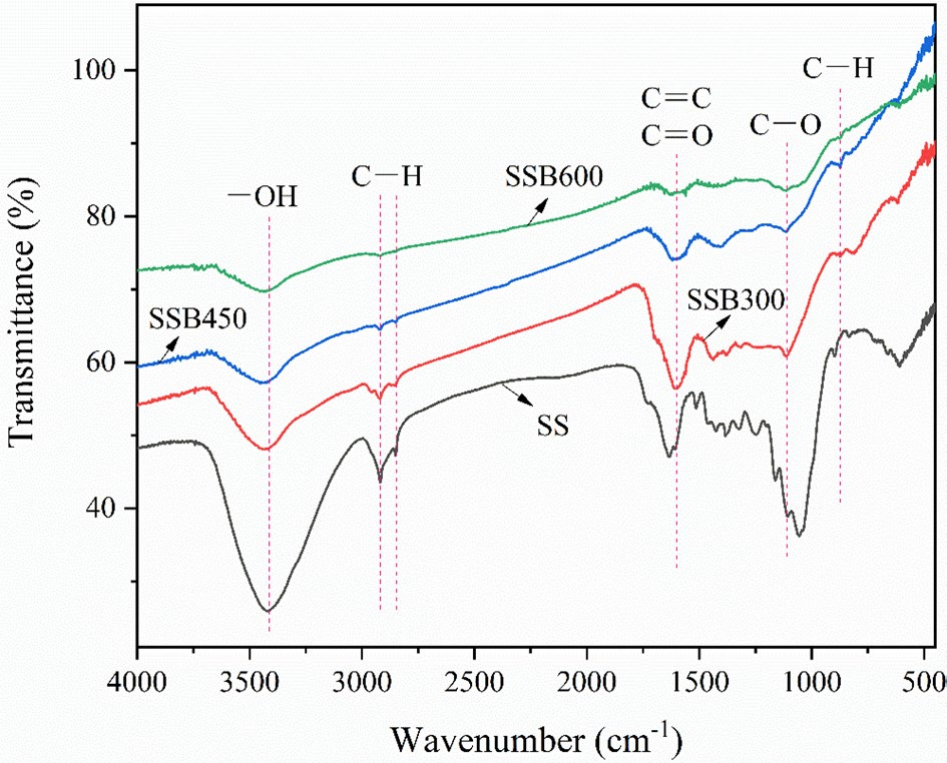
Infrared spectra of sorghum straw and its biochar.

The infrared spectrum of SS and SSB are showed in Fig. 2. The absorption peak near 3417 cm^−1^ is the –OH stretching vibration absorption peak^42^. The absorption peak gradually decreases with the increase of carbonization temperature. The increase of carbonization temperature improves the loss of hydroxyl during the pyrolysis process, thereby reducing the polarity of biochar. The absorption peaks near 2852 cm^−1^ and 2919 cm^−1^ are C–H symmetry and anti-symmetric stretching vibration absorption peaks of the methyl group in the alkyl group and the methylene group^43^. The absorption peak gradually decreases with the increase of carbonization temperature, proving that carbonization temperature is proportional to the aromaticity of biochar. The absorption peaks near 1714 cm^−1^ and 1100 cm^−1^ are the C=O in the carboxyl group and the CO stretching vibration absorption peak in the ester group^44^. As the carbonization temperature increases, these absorption peaks gradually decreases, proving that the polarity of biochar gradually decreases. The absorption peak near 1596 cm^−1^ is the stretching vibration absorption peak of C=C on the aromatic ring and C=O in the carbonyl group^45^. As the carbonization temperature increases, the peak gradually weakens. It may be that the carbonyl group in biochar is destroyed by high temperatures to generate CO and CO_2_^46^ The absorption peak near 875 cm^−1^ is the C–H flexural vibration absorption peak on the aromatic ring^47^. The absorption peak gradually increases with the increase of carbonization temperature, indicating that the stability and aromaticity of biochar are stronger. Therefore, from the analysis of infrared spectroscopy, the biochar contains a lot of oxygen-containing functional groups such as OH, COOH, and C=O, which gradually decreases as the carbonization temperature increases. The biochar pyrolyzes at low temperature is more conducive to adsorption, which is consistent with the results of oxygen-containing functional groups.

### Adsorption influencing factors

#### Carbonization temperature

**Figure 3:**
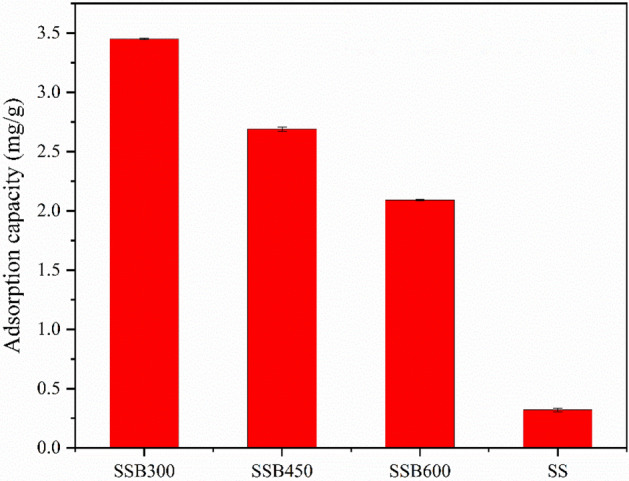
The adsorption capacity of biochar and sorghum straw for ammonium at different carbonization temperatures.

From Fig. 3, SSB300 had the highest adsorption capacity for ammonium, and the adsorption capacity and adsorption rate were 3.45 mg/g and 16.8%, respectively. SS had the smallest adsorption capacity (0.321 mg/g). Overall, the adsorption capacity was in the order of SSB300 > SSB450 > SSB600 > SS. As carbonization temperature increased, the adsorption of ammonium by SSB had a downward trend. SS also had certain adsorption of ammonium, but its adsorption performance was much lower than SSB. Although SSB300 had the smallest SSA, it had the largest content of acidic oxygen-containing functional groups and stronger ion exchange capacity, which made the SSB300 had the largest adsorption of ammonium in water.

#### Adsorbent dosage

**Figure 4:**
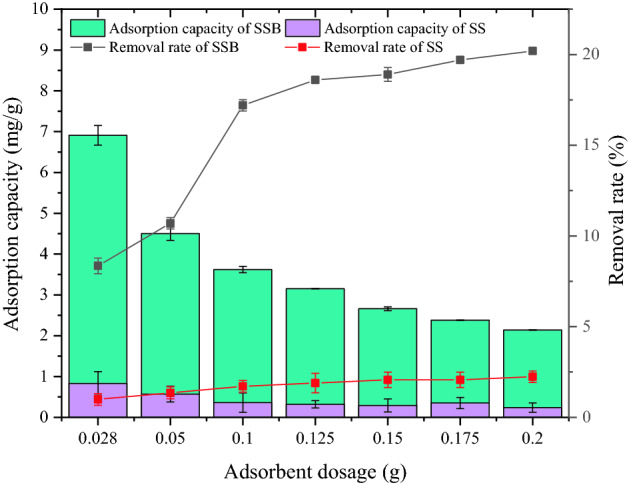
The effect of dosage on ammonium adsorption.

As shown in Fig. 4, with an increase of dosage, the adsorption capacity of SSB300 and SS for ammonium showed a downward trend, while the adsorption rates were upward. It was because as the amount of adsorbent increased, the SSA and adsorption pores of the adsorbent increased, thereby increasing the adsorption rate of ammonium. When the dosage of adsorbent was less than 0.1000 g, the ammonium adsorption rates of SSB300 and SS rose fast. When it exceeded 0.1000 g, the increasing trend was gentle, which indicated that more adsorbents can’t effectively adsorb the ammonium in the water. Therefore, 0.1000 g was chosen as the optimal dosage for the test of SS and SSB to adsorb ammonium in water.

#### Solution pH

**Figure 5:**
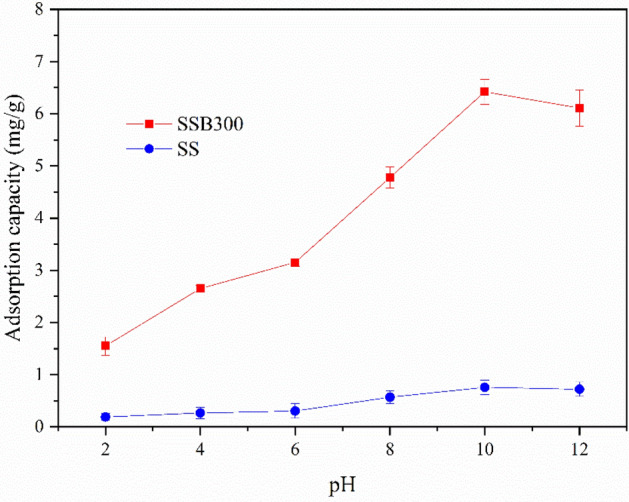
The effect of pH on ammonium adsorption.

The adsorption effect of ammonium is greatly affected by pH^48^. As shown in Fig. 5, when pH < 10, the ammonium adsorption capacity of SSB300 and SS rose with the increase of pH. Especially when the pH was 6–10, the adsorption capacity improved fast. When pH > 10, the adsorption of ammonium onto SSB300 and SS gradually decreased. The analysis believed that under acidic conditions, H^+^ facilitated the development of NH_3_·H_2_O ionization equilibrium toward NH_4_^+^, thereby enhancing the adsorption of ammonium. But as the pH decreased, too much H^+^ in the solution would bring competition to the adsorption of NH_4_^+^, inhibiting the adsorption of ammonium^35^. Under strong alkaline conditions (pH > 10), too much OH^−^ inhibited the ionization balance of NH_3_·H_2_O, resulting in a decrease of NH_4_^+^ in the solution, thereby reducing the adsorption of ammonium. In addition, when pH > 10, a part of ammonium volatilized in gaseous form. Therefore, the optimal pH of SSB300 and SS for ammonium adsorption was in the range of neutral and slightly acidic to slightly alkaline (6 ~ 10). Considering the uniformity of the subsequent actual sewage treatment control test conditions, pH = 7 was selected for subsequent experiments.

### Adsorption kinetics

**Figure 6:**
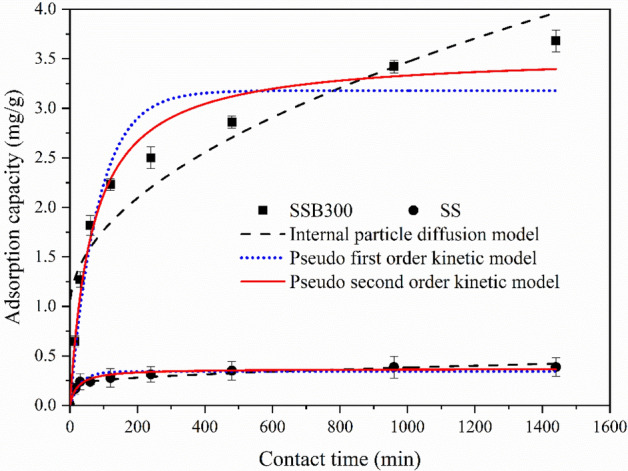
Ammonium adsorption kinetics fitting curve.

The intraparticle diffusion model, the pseudo-first-order kinetic model and the pseudo-second-order kinetic model were used to fit the experimental data. The equations were shown in (1) ~ (3). The fitting curve was shown in Fig. 6, and the fitting parameters were shown in Table 2.

1 $${\text{Intraparticle diffusion model}}:q_{t} = k_{1} t^{1/2} + C$$

2 $${\text{Pseudo-first-order kinetic model: }}q_{t} = q_{e} (1-e^{-kt}_{2})$$

3 $${\text{Pseudo-second-order kineticmodel}}:\frac{t}{{q_{t} }} = \frac{1}{{k_{3} q_{e}^{2} }} + \frac{t}{{q_{e} }}$$

where *q*_*t*_ represents the adsorption capacity of ammonium at time t, mg/g. *q*_*e*_ is the adsorption capacity of ammonium at adsorption equilibrium, mg/g. *k*_*1*_*, k*_*2*_*, k*_*3*_ are adsorption rate constants; *C* is a constant.

**Table 2:** Fitting parameters of ammonium adsorption kinetics.

Adsorbents	Intraparticle diffusion model			Pseudo-first-order kinetic model			Pseudo-second-order kinetic model		
	C	k_1_ (mg·g^−1^·min^−0.5^)	R^2^	q_e_ (mg·g^−1^)	K_2_ min^−1^	R^2^	q_e_ (mg·g^−1^)	K_3_ (mg·g^−1^·min^−1^)	R^2^
SSB300	0.9830	0.0786	0.8718	3.1786	0.0121	0.9218	3.5529	0.0042	0.9733
SS	0.1960	0.0059	0.8490	0.3416	0.0329	0.8758	0.3694	0.1208	0.9526

As shown in Table 2 and Fig. 6, the correlation coefficient *R*^2^ of the pseudo-second-order kinetic model was the highest, and the *R*^2^ of SSB300 was higher than that of SS. The theoretical equilibrium adsorption capacities were 3.55 and 0.375 mg/g respectively, which were very close to the actual saturated adsorption capacities (3.68 and 0.388 mg/g). Therefore, the pseudo-second-order kinetic model was more consistent with the adsorption process of SS and SSB300 on ammonium in water.

From the perspective of the particle diffusion model, the adsorption process of SS and SSB300 on ammonium in water could be roughly divided into two stages. The first 3 h was the process of rapid adsorption of ammonium onto the surface of the adsorbent. Phase 2: After 3 h ammonium diffused slowly into the adsorbent. Due to the influence of molecular diffusion resistance, the diffusion rate within the adsorption decreased until the adsorption tended to balance. This stage was the rate-controlling stage of ammonium adsorption. Due to *C* ≠ 0, it indicated that intra-particle diffusion was not the only speed control step, meaning that the fitting curve was not at the origin^49^. The adsorption rate might be controlled by surface adsorption and intra-particle diffusion^50,51^.

### Adsorption isotherms

**Figure 7:**
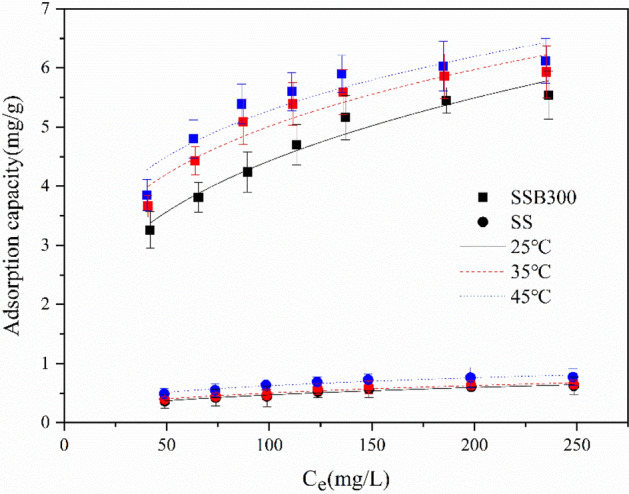
The adsorption thermodynamic isotherm of ammonium (Freundlich).

**Figure 8:**
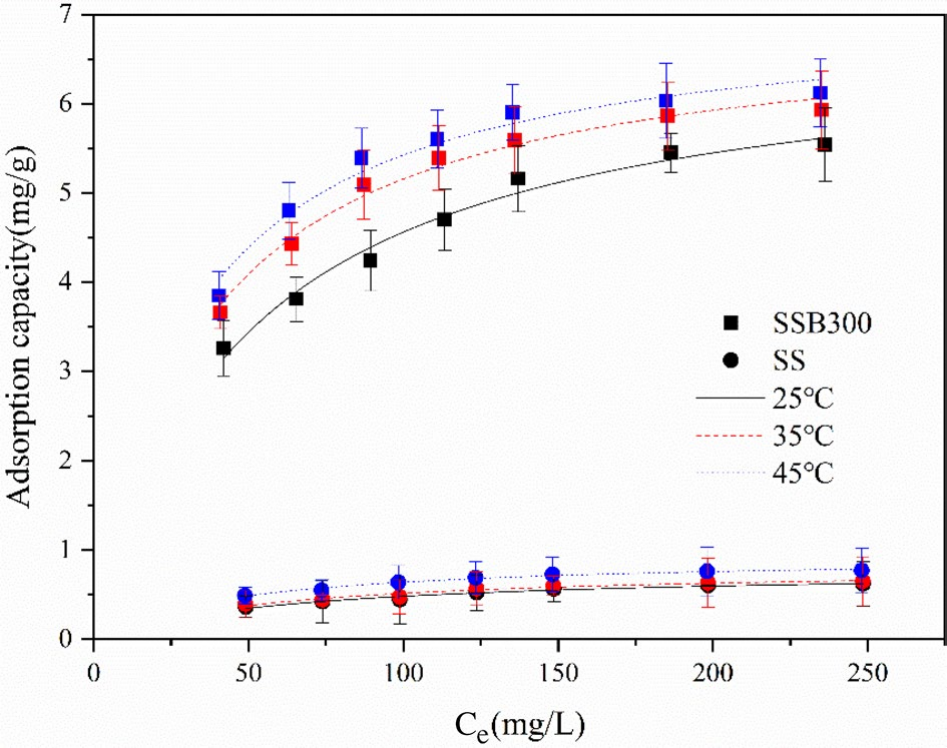
The adsorption thermodynamic isotherm of ammonium (Langmuir).

The Freundlich and Langmuir isotherm adsorption models were fitted to the adsorption test data at 25, 35, and 45 °C. The fitting curves were shown in Figs. 7 and 8, respectively, and the fitting parameters were shown in Table 3. The equations of the Freundlich and Langmuir isotherm adsorption models were as follows:

4 $${\text{Freundlich model}}:q_{e} = k_{F} C_{e}^{1/n}$$

**Table 3:** Isothermal adsorption fitting parameters of ammonium.

Adsorbents	T(K)	Freundlich model			Langmuir model			
		K_F_ (L/mg)	1/n	R^2^	q_m_ (mg/g)	K_L_ (L/mg)	R^2^	R_L_
SSB300	298	1.0553	0.3113	0.9541	6.7655	0.0207	0.9779	0.162 ~ 0.491
	308	1.5630	0.2530	0.8979	6.9687	0.0284	0.9838	0.123 ~ 0.423
	318	1.8252	0.2306	0.8599	7.0930	0.0325	0.9699	0.110 ~ 0.381
SS	298	0.0968	0.3431	0.9539	0.7817	0.0161	0.9608	0.199 ~ 0.544
	308	0.1135	0.3234	0.9339	0.8068	0.0177	0.9458	0.184 ~ 0.531
	318	0.1703	0.2824	0.9295	0.9336	0.0218	0.9785	0.155 ~ 0.478

5 $$\text{Langmuir model}: {q}_{e}=\frac{{q}_{m}{k}_{L}{C}_{e}}{1+{k}_{L}{C}_{e}}$$

In the above formula, *q*_*m*_ is the theoretical maximum adsorption capacity of ammonium, mg/g. *C*_*e*_ is the concentration of ammonium in the solution at adsorption equilibrium, mg/L. *k*_*F*_*, k*_*L*_*, n* are isotherm constants.

As shown in Table 3, Figs. 7 and 8, the correlation coefficient *R*^2^ of the Langmuir isotherm adsorption model was higher than that of the Freundlich isotherm adsorption model, proving that the Langmuir isotherm adsorption model was more in line with the thermodynamic adsorption behavior of SS and SSB300 for ammonium in water. And they were mainly monolayer adsorption. At 25, 35, and 45 °C, the maximum adsorption capacity of SSB300 for ammonium was 6.77, 6.97, and 7.09 mg/g, respectively, which was equivalent to 8.65, 8.64, and 7.60 times of SS. Compared with other adsorbents, SSB300 in this study showed a high ammonium adsorption capacity (Table 4). This difference was mainly attributed to the differences in physical and chemical properties between different biochar.

**Table 4:** A comparison of the adsorption capacities of other adsorbents for ammonium.

Biochars	Adsorption capacity (mg/g)	References
Wood chips biochar	0.96	^52^
Sludge biochar	1.2	^53^
Bamboo biochar	7.0	^54^
Coffee husk biochar	2.8	^55^
Canna biochar	5.6	^29^
Corn stalk biochar	7.174	^30^
Sorghum straw biochar	7.09	This study

In addition, the separation factor *R*_*L*_ was used to judge the adsorption effectiveness of the adsorbent^56^. The equation was:

6 $${\text{Separation factor}: R}_{L}=\frac{1}{1+{k}_{L}{C}_{0}}$$

*C*_*0*_–The initial concentration of ammonium in the solution, mg/L.

Among them, *R*_*L*_ = 0 is irreversible adsorption. 0 < *R*_*L*_ < 1 is favorable adsorption. *R*_*L*_ = 1 is linear adsorption.

From Table 3, the *R*_*L*_ of SS and SSB300 at the three temperatures was all greater than 0 but less than 1, indicating that the SS and SSB had favorable adsorption of ammonium in water.

### Adsorption thermodynamics

Judging from the isotherm adsorption models of SS and SS300 at 3 temperatures, the adsorption capacity gradually increased as the temperature improved. To further study the thermodynamic behavior of adsorption, the Gibbs free energy change, enthalpy change and entropy change were analyzed according to the Langmuir isotherm adsorption model parameters in Table 3. The calculation formula was as follows:

7 $$\Delta G^{\theta } = - RT\ln K_{L}^{\theta } = \Delta H^{\theta } - TS^{\theta }$$

8 $$\ln K_{L}^{\theta } = \frac{{\Delta S^{\theta } }}{R} - \frac{{\Delta H^{\theta } }}{RT}$$

In the above formula, *△G*^*θ*^ is Gibbs free energy change, kJ/mol. *△H*^*θ*^ is enthalpy change, kJ/mol. *△S*^*θ*^ is entropy change, kJ/mol. *R* is gas constant, J/mol·K. *K*_*L*_^*θ*^ is the standard equilibrium constant, that is, the Langmuir isotherm adsorption model empirical equilibrium constant after standard concentration correction, dimensionless.

The Gibbs free energy change, enthalpy change and entropy change calculated by formulas (7) and (8) are shown in Table 5.

**Table 5:** The adsorption thermodynamic parameters of ammonium.

Adsorbents	T(K)	Langmuir model		△G^θ^ (kJ/mol)	△H^θ^ (kJ/mol)	△S^θ^ (kJ/mol·K)
		K_L_ (L/mg)	K_L_^θ^			
SSB300	298	0.0207	3067.8^a^	− 19.89	− 24.03	− 0.1417
	308	0.0284	2025.4^a^	− 18.86		
	318	0.0325	1670.4^a^	− 18.39		
SS	298	0.0161	2.98 × 10^5a^	− 31.23	− 26.76	0.1553
	308	0.0177	2.54 × 10^5a^	− 30.83		
	318	0.0218	1.50 × 10^5a^	− 29.54		

^a^When calculating the molar concentration of the adsorbent, the molecular weights of SS300 and SS were assumed to be the molecular weight of C (12 g/mol).

From the results in Table 5, *△G*^*θ*^ < 0, showed that the adsorption process of SS and SSB to ammonium in water was spontaneous. The *△H*^*θ*^ of SS and SS300 were both less than 0, indicating that the adsorption process of SS and SSB to ammonium in water was exothermic.

### Adsorption mechanisms

According to the results of BET, FTIR and SEM analysis of SSB, the biochar with higher carbonization temperature had higher SSA and pore volume. However, adsorption results showed that biochar with low carbonization temperature had the best adsorption effect on ammonium in water. Through the analysis of the oxygen-containing functional group test results and the FTIR diagram of the biochar, it was found that the biochar with lower carbonization temperature contained more oxygen-containing functional groups, which was more conducive to the adsorption of ammonium. This analysis result was consistent with the actual adsorption results. It proved that the adsorption of SSB to ammonium in water was not simply physical adsorption, but a complex physical and chemical adsorption process dominated by chemical adsorption^51^. From the perspective of adsorption kinetics, the adsorption process was more in line with pseudo-second-order kinetics, which was consistent with the mechanism of clay/biochar composite adsorption particles on ammonium^33^. From the perspective of isothermal adsorption, the Langmuir isotherm adsorption model was more consistent with the adsorption behavior of SS and SSB on ammonium in water, which was consistent with the adsorption mechanism of food waste-based biochar on ammonium in water^30^. The thermodynamic analysis proved that the adsorption process was mainly chemical adsorption, which was a spontaneous and exothermic process.

### Regeneration performance

**Figure 9:**
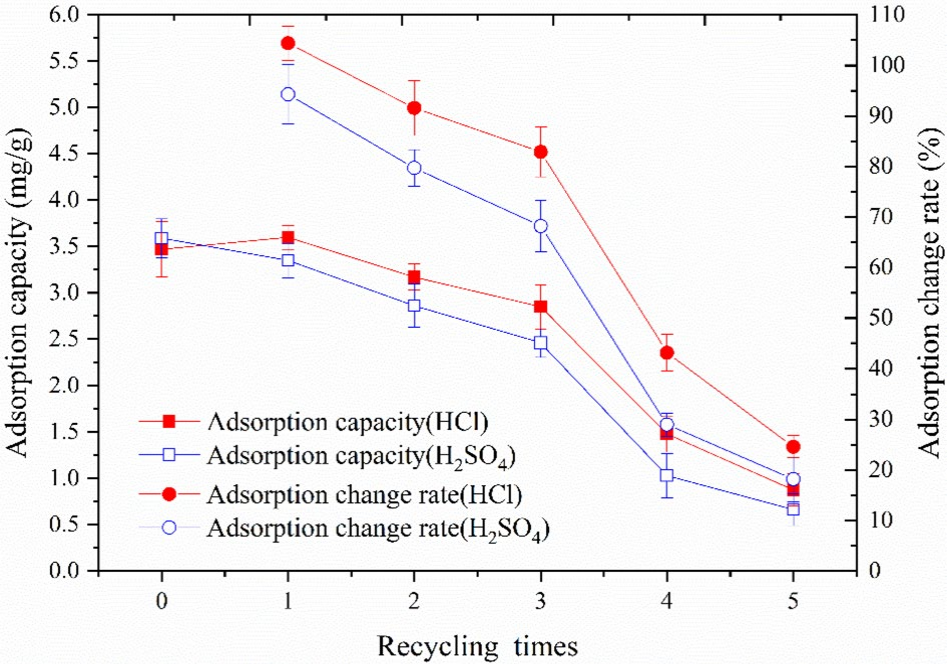
Adsorption effect under different regeneration conditions.

To investigate the regeneration performance of SS and SS300 after adsorbing ammonium, they were regenerated with HCl and H_2_SO_4_ solutions respectively. The adsorption effect after regeneration was shown in Fig. 9. The adsorption capacity of SS and SS300 after regeneration by HCl solution was higher than that of H_2_SO_4_ solution. With repeated experiments of adsorption-regeneration, the first 3 adsorption-regeneration cycles of SS300 changed slightly. It was determined that the optimal number of regenerations of SS300 under the condition of HCl solution was 3, and the equilibrium adsorption capacity after regeneration was 82.1% of the initial adsorption capacity. Since the adsorption capacity of SS was very small, the adsorption capacity became extremely low after the first adsorption-regeneration cycle. Therefore, the regeneration performance of SS after adsorbing ammonium was extremely low, while the SSB had good regeneration performance after adsorbing ammonium.

## Conclusions

SS and SSB can adsorb ammonium well in water. The adsorption of ammonium onto SSB is much higher than that of SS. The favorable pH for ammonium adsorption is 6–10 and the optimal dosage is 2.5 g/L. Low carbonization temperature (300 °C) is more conducive to the formation of functional groups, which are beneficial to the adsorption of ammonium. The adsorption process of SS and SSB to ammonium in water is more in line with the pseudo-second-order kinetic model. The adsorption behavior is more in line with the Langmuir isotherm adsorption model. The maximum ammonium adsorption capacity at 25, 35, and 45 °C are 6.77, 6.97, and 7.09 mg/g, which are equivalent to 8.65, 8.64, and 7.60 times of SS, respectively. And the adsorption process is spontaneous and exothermic. The study indicates that SSB can be applied to sewage treatment containing ammonium to achieve the purpose of resource recycling.

## Supplementary Information

Supplementary Information.
