# Supplementary material for: Adsorption behavior and performance of ammonium onto sorghum straw biochar from water
Source: Sci Rep. 2022 Mar 30;12:5358. doi: 10.1038/s41598-022-08591-5 (PMC8967861; doi:10.1038/s41598-022-08591-5)
Supplement: Supplementary file 1 — Supplementary Information. [file 41598_2022_8591_MOESM1_ESM.docx]

**Statement**

For reasons of scientific research, sorghum straws used in this study were collected from the organic sorghum base in suburb of Renhuai City, Guizhou Province, China. The sampling location is near the first author's institute which belongs to a private farmland. We have obtained the permission from the land owner to sample sorghum straws for scientific research.
